# In depth sequencing of a serially sampled household cohort reveals the within-host dynamics of Omicron SARS-CoV-2 and rare selection of novel spike variants

**DOI:** 10.1371/journal.ppat.1013134

**Published:** 2025-04-28

**Authors:** Emily E. Bendall, Derek Dimcheff, Leigh Papalambros, William J. Fitzsimmons, Yuwei Zhu, Jonathan Schmitz, Natasha Halasa, James Chappell, Emily T. Martin, Jessica E. Biddle, Sarah E. Smith-Jeffcoat, Melissa A. Rolfes, Alexandra Mellis, H. Keipp Talbot, Carlos Grijalva, Adam S. Lauring

**Affiliations:** 1 Department of Microbiology & Immunology, University of Michigan, Ann Arbor, Michigan, United States of America; 2 Department of Internal Medicine, University of Michigan, Ann Arbor, Michigan, United States of America; 3 Department of Biostatistics, Vanderbilt University Medical Center, Nashville, Tennessee, United States of America; 4 Department of Pathology, Vanderbilt University Medical Center, Nashville, Tennessee, United States of America; 5 Department of Pediatrics, Vanderbilt University Medical Center, Nashville, Tennessee, United States of America; 6 Department of Epidemiology, University of Michigan, Ann Arbor, Michigan, United States of America; 7 Centers for Disease Control and Prevention, Atlanta, GeorgiaUnited States of America; 8 Department of Health Policy, Vanderbilt University Medical Center, Nashville, Tennessee Tennessee, United States of America; 9 Department of Medicine, Vanderbilt University Medical Center, Nashville, Tennessee, United States of America; Emory University School of Medicine, UNITED STATES OF AMERICA

## Abstract

SARS-CoV-2 has undergone repeated and rapid evolution to circumvent host immunity. However, outside of prolonged infections in immunocompromised hosts, within-host positive selection has rarely been detected. Here we combine daily longitudinal sampling of individuals with replicate sequencing to increase the accuracy of and lower the threshold for variant calling. We sequenced 577 specimens from 105 individuals in a household cohort during the BA.1/BA.2 variant period. Individuals exhibited extremely low viral diversity, and we estimated a low within-host evolutionary rate. Within-host dynamics were dominated by genetic drift and purifying selection. Positive selection was rare but highly concentrated in spike. A Wright Fisher Approximate Bayesian Computational model identified positive selection at 14 loci with 7 in spike, including S:448 and S:339. This detectable immune-mediated selection is unusual in acute respiratory infections and may be caused by the relatively narrow antibody repertoire in individuals during the early Omicron phase of the SARS-CoV-2 pandemic.

## Author summary

Throughout the SARS-CoV-2 pandemic new variants have continually arisen due to population level immunity from vaccinations and previous infections. A similar process within-hosts may be occurring, where new variants evolve during the course of an infection to escape partial immunity from previous exposures. However, within-host positive selection has rarely been detected in acute SARS-CoV-2 infections. Here we studied individuals from a case-ascertained household cohort during the BA.1/BA.2 wave. Nasal swab specimens were collected daily for 10 days and sequenced in replicate for high accuracy. Individuals exhibited extremely low viral diversity with stochastic processes dominating. Positive selection was rare but highly concentrated in spike. The majority of spike mutations we identified as positively selected have been implicated in immune evasion previously. This infrequent but detectable positive selection may be due to the timing of these infections relative to the emergence of SARS-CoV-2

## Introduction

As SARS-CoV-2 continues to circulate, population immunity from infections and vaccinations has resulted in the evolution of new variants that quickly become the dominant circulating strain [1,2]. This has contributed to decreased vaccine effectiveness, and in response, multiple reformulations of the SARS-CoV-2 vaccines [3–5]. The continual evolution of SARS-CoV-2 as a result of selection from the host adaptive immune system is likely to continue. Similar to this global antigenic drift, partial immunity from previous exposure may lead to the selection of new antigenic variants within hosts [6,7]. Because all variation originates from intrahost processes, understanding within-host dynamics is crucial to understanding the evolutionary trajectory of SARS-CoV-2.

To date, there has been limited evidence of positive selection of immune escape variants within individuals with acute, self-limited SARS-CoV-2 infections. We and others have found that SARS-CoV-2 infections exhibit low genetic diversity and few *de novo* mutations that reach significant frequencies [8–11]. Select studies have identified spike variants in sites known to confer antibody resistance [8,11]. Additionally, Farjo *et al.* found nonsynonymous intrahost single nucleotide variants (iSNVs) to be enriched in individuals who had been vaccinated or previously infected [11]. Regions of within-host positive selection in non-spike regions have also been detected when comparing intrahost diversity of synonymous and nonsynonymous variants (p_N_/p_S_) [12]. However, genetic hitchhiking (i.e., changes in a mutation’s frequency as a result of selection on a linked site on the same genome/chromosome) and genetic drift make it difficult to accurately detect positive selection with viruses from only a single timepoint [13].

Most studies of serially sampled individuals come from prolonged infections in immunocompromised patients, where immune escape variants have repeatedly been found [14–18]. Prolonged infections release the virus from the frequent population bottlenecks characteristic of acute infections, increasing the amount of genetic variation and allowing time for selection to occur [19]. The selection pressures in immunocompromised individuals may differ from those in immunocompetent individuals with acute infections, with selection for increased cell-cell transmission and viral packaging [17]. Additionally, monoclonal antibodies commonly used to treat immunocompromised individuals may exert more targeted selection than a polyclonal response from prior exposure in immunocompetent individuals [20].

To more thoroughly examine the role of positive selection within hosts during acute SARS-CoV-2 infections, we studied individuals from a case-ascertained household cohort, in which nasal swab specimens were collected daily for 10 days after enrollment. All specimens were sequenced in duplicate, allowing for robust variant calling at a very low frequency threshold (0.5%). With serial sampling and low frequency variant calling, we were able to define the within-host divergence of SARS-CoV-2 populations, detect genetic hitchhiking, and identify rare, but potentially significant, instances of positive selection in spike.

## Methods

### Cohort and specimens

Households were enrolled through the CDC-sponsored Respiratory Virus Transmission Network – Sentinel (RVTN-S), a case ascertained household transmission study coordinated at Vanderbilt University Medical Center [21]. All individuals provided written, informed consent and parents/guardians provided written, informed consent for minors. Individuals included in the current study were enrolled in Nashville, TN from September 2021 to February 2022. The study was reviewed and approved by the Vanderbilt University Medical Center Institutional Review Board (see 45 C.F.R. part 46.114; 21 C.F.R. part 56.114). Index cases (i.e., the first household members with laboratory-confirmed SARS-CoV-2 infection) were identified and recruited from ambulatory clinics, emergency departments, or other settings that performed SARS-CoV-2 testing. Index cases and their households were screened and enrolled within 6 days of the earliest symptom onset date within the household. Vaccination status was determined by plausible self-report (report of a manufacturer and either a date or location) or vaccine verification through vaccination cards, state registries, and medical records. Only vaccines received more than 14 days before the date of the earliest symptom onset in the household were considered.

Nasal swabs specimens were self- or parent-collected daily from all enrolled household members during follow-up for 10 days and tested for SARS-CoV-2. Nasal swabs were tested by transcription mediated amplification using the Panther Hologic system. All available specimens were processed for sequencing as described below.

### Sequencing and variant calling

SARS-CoV-2 positive specimens with a cycle threshold (Ct) value ≤32 were sequenced in duplicate after the RNA extraction step. RNA was extracted using the MagMAX viral/pathogen nucleic acid purification kit (ThermoFisher) and a KingFisher Flex instrument. Sequencing libraries were prepared using the NEBNext ARTIC SARS-CoV-2 Library Prep Kit (NEB) and ARTIC V5.3.2 primer sets. After barcoding, libraries were pooled in equal volume. The pooled libraries (up to 96 specimens per pool) were size selected by gel extraction and sequenced on an Illumina NextSeq (2x300, P1 chemistry).

For the first specimen per individual with adequate sequencing, we aligned the sequencing reads to the MN908947.3 reference using BWA-mem v0.7.15 [22]. Primers were trimmed using iVar v1.2.1 [23]. Reads from both replicates were combined and used to make a within host consensus sequence using a script from Xue et al [24]. Specimens were considered successfully sequenced if both replicates had an average genome wide coverage > 1000x. All specimens were aligned to their respective within-host consensus sequences. Primers were trimmed using iVar and reads from amplicons with mismatched primers were masked. Intrahost single nucleotide variants (iSNV) were identified for each replicate separately using iVar [23] with the following criteria: frequency 0.005-0.995, p-value < 1x10^-5^, variant position coverage depth > 400x. We also masked ambiguous and homoplastic sites that have previously been designated as probably erroneous [25]. Specific to this study, T11075C was found at low frequencies in 48 individuals. This is indicative of a sequencing artifact, and T11075C was also masked. Finally, to minimize the possibility of false variants being detected, the variants had to be present in both sequencing replicates. The variant frequencies were averaged for all analyses. Indels were not evaluated. Lineages were determined with Nextclade [26] and Pango [27,28], based on the within-host consensus sequence.

### iSNV Dynamics and divergence rates

We calculated the divergence rate as in Xue *et al* [24]. Briefly, we calculated the rate of evolution by summing the frequencies of within-host mutations (non-consensus allele in first specimen) and dividing by the number of available sites and the time since the infection began. We calculated the rates separately for nonsynonymous and synonymous mutations. We used 0.77 for the proportion of available sites for nonsynonymous mutations and 0.23 for synonymous. To determine the number of available sites, we multiplied the proportion of sites available by the length of the coding sequence of the MN908947.3 reference. Because symptoms typically start 2–3 days post infection and nasal swab collection occurred after symptom onset among most individuals, we added 2 days to the time since symptom onset to obtain the time elapsed between infection and sampling [29–31]. We excluded individuals who were asymptomatic from the divergence rate analysis, as we are not able to date their infection by symptom onset (e.g., 2–3 days prior as above). Because the calculated rate of divergence varied over the course of the infection, we also calculated the rate using the specimen with the highest viral load for each individual to control for timing within the course of the infection (S1 Fig). In addition, we used linear regression to estimate the divergence rates in individuals with multiple specimens. We calculated per-site viral divergence for each specimen. For each person, a linear regression was performed with the per specimen divergences and the days post infection with viral load as a covariate. A person’s divergence rate was the slope of this regression line. The rate was calculated for the whole genome and for each gene separately.

Mann-Whitney U tests were used to determine if the number iSNV per specimen and iSNV frequencies differed by mutation type, vaccination, and age group. Kruskal-Wallace tests were performed to determine if the number iSNV per specimen and iSNV frequencies differed by clade and days post symptom onset. Generalized linear models were used to determine if viral load impacted iSNV frequency and iSNV count (Poisson distributions). Mann-Whitney U tests were used to determine if the divergence rate differed by vaccination and age group. Kruskal-Wallace tests were performed to determine if divergence rate differed by clade, gene and days post infection. A generalized linear model was used to determine if viral load impacted divergence rates. All analyses were conducted using R version 4.3.1.

### Analysis of selection

The study period included the Delta, BA.1, and BA.2 variant periods of the SARS-CoV-2 pandemic. For each of these clades, we looked at the lineage-defining mutations in spike of the subsequent wave (i.e., BA.1, BA.2, and BA.4/BA.5). We compared the iSNV within our specimens to these lineage defining mutations.

We also used Wright Fisher Approximate Bayesian Computation (WFABC) to estimate the effective populations size (Ne) and per locus selection coefficient (s) based on allele trajectories [32]. Generation times of 8 hours and 12 hours were used [33–35]. To maximize the number of loci used in the calculation of Ne and to avoid violating the assumption that most loci are neutral, we estimated a single Ne using all loci from individuals in which the first two specimens sequenced were collected one day apart. 10,000 bootstrap replicates were performed to obtain a posterior distribution. A fixed Ne was used for the per locus selection coefficient simulations, with the analysis repeated for the mean Ne, and the + /- 1 standard deviation Ne estimated from the previous step. A uniform prior between s of -0.5 and 0.5 was used with 100,000 simulations and an acceptance rate of 0.01. We estimated the 95% highest posterior density intervals using the boa package [36] in R. We considered a site to be positively selected if the 95% highest posterior density did not include 0 for all three effective population sizes.

To understand how within-host selection relates to between host selection, we used the SARS-CoV-2 Nextstrain build [37] (nextstrain/ncov, the Nextstrain team) to examine the global frequencies of iSNV that were under positive within-host selection in our study. We also compared the selection coefficients we estimated to the selection coefficients that Bloom and Neher [38] estimated from the global phylogeny.

## Results

**Fig 1:**
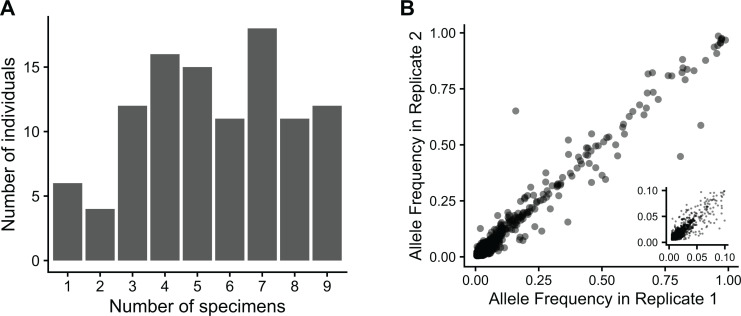
Sequencing summary. **(A)** The number of specimens per person successfully sequenced. **(B)** intra-host single nucleotide variants (iSNV) frequency is consistent across replicates for iSNV that pass quality control filtering. The insert shows iSNV frequency up to 0.1.

There were 212 SARS-CoV-2 infected individuals enrolled from September 2021 to February 2022 in this case-ascertained household cohort. None of the individuals enrolled received monoclonal antibodies or antivirals. Of these, we successfully sequenced 577/825 (70%) specimens from 105 individuals. Ninety-nine out of 105 (94%) individuals had multiple specimens successfully sequenced (Fig 1A, S1 Table). Consistent with the viruses circulating in the United States during this timeframe, the individuals in the study were infected with Delta, BA.1, and BA.2. Depth of coverage was generally high (S2 Fig) and iSNV frequency was similar between replicates (Fig 1B). The number of iSNV detected was weakly related to sequencing depth with an adjusted R^2^ of 0.04 (S2 Fig, t = 5.201, p = 2.77x10^-7^).

### iSNV dynamics

**Fig 2:**
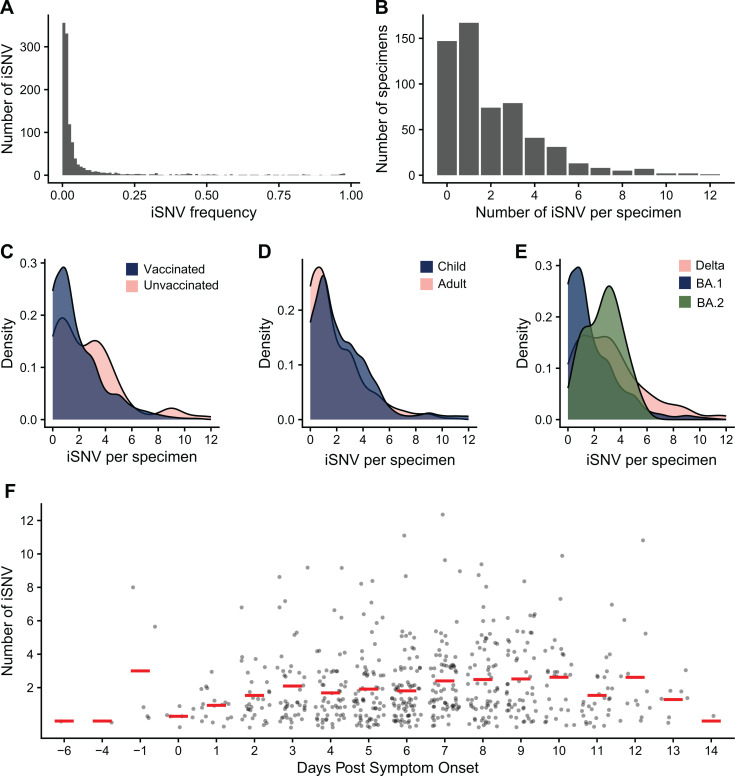
Intrahost single nucleotide variant dynamics. **(A)** iSNV frequency. **(B)** The number of iSNV per specimen. The number of iSNV per specimen by **(C)** vaccination status, **(D)** age with child <18 and adult 18 + , **(E)** clade, **(F)** and days post symptom onset. The red lines are the mean. Vaccinated individuals and BA.1 infections had fewer iSNV per specimen than unvaccinated individuals and Delta or BA.2 infections.

The allele frequencies of identified iSNV were generally very low, with the majority of iSNV present at ≤ 2% frequency (Fig 2A). In our cohort, the frequencies of iSNV in vaccinated individuals (median = 0.0151) were higher than in unvaccinated individuals (median = 0.0127, p = 0.022, S2 Table), but this difference was extremely small and unlikely to be biologically significant (S3 Fig). Frequencies of iSNV also varied by the day of sampling (p = 0.002, S3 Fig, S2 Table) but did not differ based on host age, SARS-CoV-2 clade, mutation type (i.e., nonsynonymous vs. synonymous; S3 Fig), or viral load (S4 Fig and S3 Table).

All specimens had between 0–12 iSNV identified at an allele frequency ≥0.5% (Fig 2B). Unvaccinated individuals (median = 2.61, p < 0.001) and children (median = 2, p = 0.011) had greater numbers of iSNV per specimen than vaccinated individuals (median = 1.82) and adults (median = 1, Fig 2C and 2D, S2 Table). BA.1 had fewer iSNV per specimen (median = 1, p < 0.001) than BA.2 (median = 3, p = 0.033) or Delta (median = 3, p < 0.001) infections (Fig 2E, S2 Table). The number of iSNV per specimen increased as the infection progressed, and after 8–10 days post symptom onset, the number of iSNV decreased (p = 0.005, Fig 2F, S2 Table). The time of sampling (days post symptom onset) did not noticeably differ by vaccination status, age, or clade (S5 Fig). The number of iSNV did not differ based on viral load (S4 Fig and S3 Table).

### Within-host divergence rates

**Fig 3:**
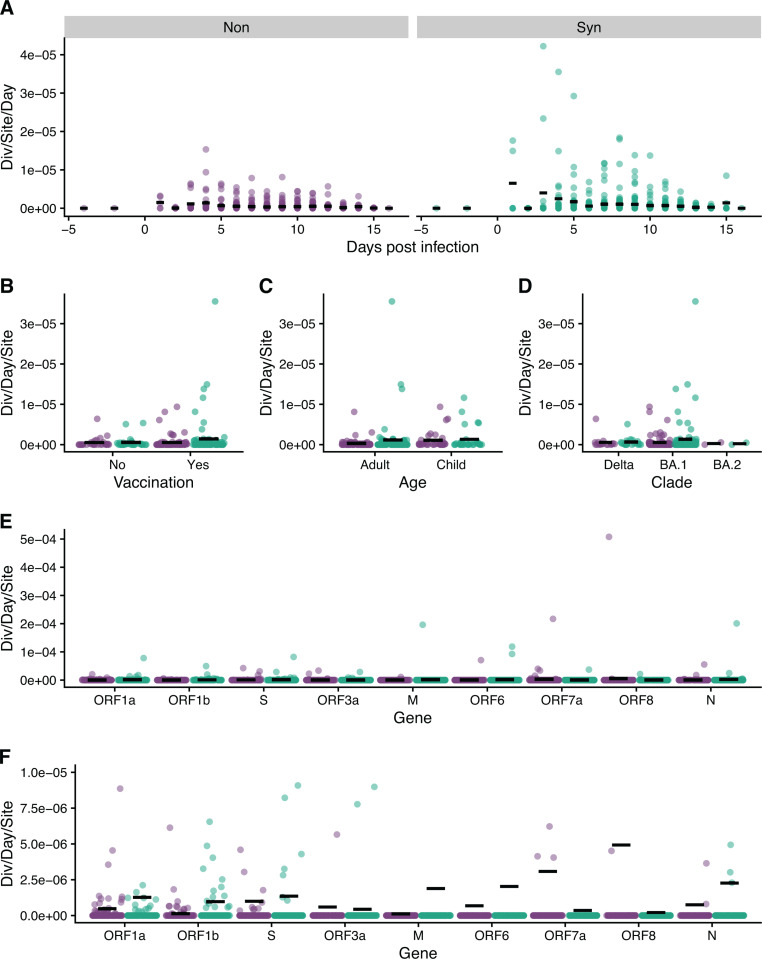
Divergence rates using a per specimen estimate. **(A)** Divergence rate (divergence/site/day) for all specimens by days post infection. Divergence rate (divergence/site/day) using the specimen with the highest viral titer by **(B)** vaccination status, **(C)** age, **(D)** clade, and **(E)** gene. **(F)** is a zoomed in version of **(E)**, note y-axis. For nonsynonymous mutations children had higher rates than adults, and spike had a higher rate than ORF1a. Black lines are the mean divergence rate. Green is synonymous, and purple is nonsynonymous.

We estimated within-host evolutionary rates as nucleotide divergence per site per day on a per-specimen basis and by linear regression in individuals for whom we had multiple sequenced specimens. The genome-wide mean divergence rate was 5.03 x 10^-7^ nucleotide substitutions/site/day for nonsynonymous mutations and 1.08 x 10^-6^ for synonymous mutations. Although not statistically significant, the estimated divergence rate varied according to the day of sampling when using a per specimen estimate (Fig 3). The divergence rate increased from the onset of the infection until approximately day 5 for nonsynonymous sites and day 8 for synonymous sites and then decreased. The divergence rate wasn’t related to viral load (S4 Fig and S3 Table).

For the rest of the comparisons using a per specimen estimate, the divergence rate from the specimen with the highest viral load was used. Children had higher rates for nonsynonymous mutations (mean = 1.11x10^-6^ vs 3.01x10^-7^), but not synonymous mutations (p = 0.019, Fig 3C, S4 Table), while rates for synonymous mutations were not associated with age. The divergence rate did not differ by vaccination status or clade (Fig 3, S4 Table). There were significant differences in divergence rate based on gene (p < 0.001); notably, spike (mean = 9.96x10^-7^) had a higher divergence rate compared to ORF1a (mean = 4.81x10^-7^) for nonsynonymous mutations, but did not differ from any of the other genes (Fig 3E and 3F, S5 Table). Results obtained by linear regression were slightly different. The divergence rate did not differ by vaccination, age, clade, or gene (S6 Fig, S4 Table). Divergence rate varied by gene for synonymous mutations (p < 0.001, S6 Fig, S4 and S6 Tables). ORF1b (mean = 2.27 x 10^-6^) had a higher divergence rate than N (mean = -2.62 x 10^-6^) and ORF8 (mean = -3.55 x 10 ^-7^). The negative estimates were due to times when specimens were collected predominantly after the peak of viral load, in a contracting population.

### Analysis of selection

We analyzed selection by first looking for iSNV that anticipated mutations that defined subsequent variants. Many lineage defining mutations are immune escape variants, and within-host selection for new antigenic variants may precede immune selection detectable at the population level as lineage defining mutations of subsequent variants. Two individuals with BA.1 had an iSNV that causes S:371F, a BA.2 lineage defining mutation (Table 1). These iSNV were at low frequencies, with a maximum observed frequency of 0.8% and 1.8%. There were 3 additional iSNV in the codon for a lineage defining mutation but resulted in a different amino acid substitution. This included a third iSNV at position 371.

**Table 1:** iSNV that are in the same position as lineage defining mutations in spike for the subsequent variant wave.

Clade	Next Wave	Lineage Defining Mut.	Observed Mutation	Individual	Max Obs. Frequency
Delta	BA.1	N440K	N440Y	102101	0.049
Delta	BA.1	G446S	G446V	101201	0.018
Delta	BA.1	T547K	T547I	101703	0.008
BA.1	BA.2	S371F	L371I	102601	0.008
BA.1	BA.2	S371F	L371F	105701	0.018
BA.1	BA.2	S371F	L371F	107303	0.008

**Fig 4:**
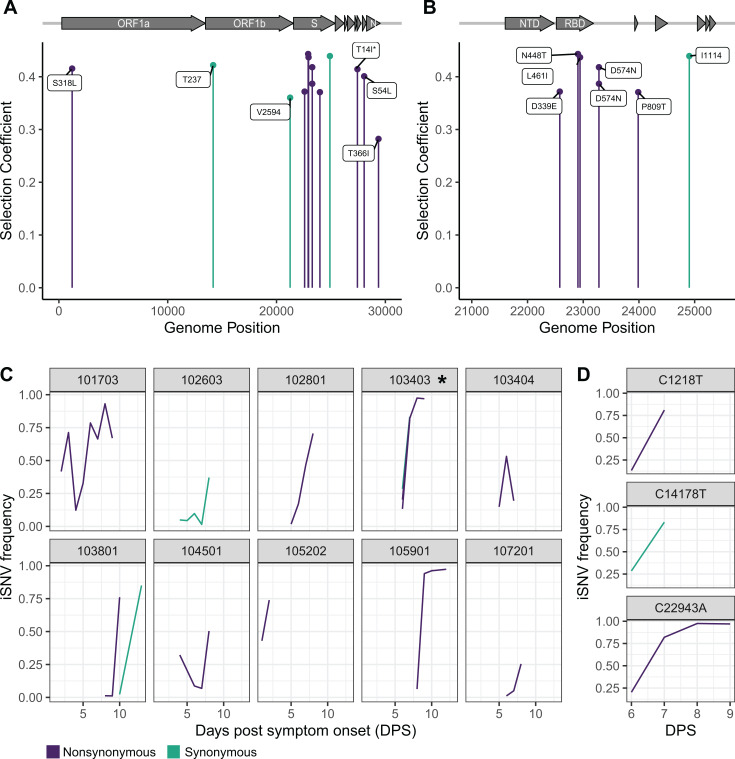
iSNV under positive selection and their corresponding allele trajectories. **(A)** WFABC selection coefficients for iSNV under positive selection for the whole genome and **(B)** for spike. **(C)** The allele trajectories of the iSNV with positive selection coefficients by individual. **(D)** The allele trajectories separated by locus for iSNV in individual 103403, denoted with an asterisk in **(C).** Green is synonymous, and purple is nonsynonymous. WFABC = Wright Fisher Approximate Bayesian Computation; iSNV = intra-host single nucleotide variants.

Using a WFABC model, we estimated a within-host effective population size of 78 resulting in strong genetic drift. Fourteen iSNV from 11 individuals were under positive selection: 7 in spike, 6 in other coding regions and 1 in a non-coding region (Fig 4A and 4B, Table 2). The results were the same for 8hr and 12hr generation times. Of the iSNV found in coding regions, 10 were nonsynonymous, including 6 of the iSNV in spike. Two of the selected synonymous iSNV were in individuals that had nonsynonymous iSNV under positive selection, suggestive of linkage as the allele trajectories of the two iSNV were closely matched (Fig 4C and 4D). Outside of spike ORF7a:T14I and ORF1a:S318L were found in 1 additional individual each. Both individuals only had a single sample and therefore no selection coefficient.

**Table 2:** iSNV with a positive selection coefficient. Selection coefficient values are from the 8hr generation time results.

Gene	iSNV	AA Mutation	Mutation Type	Selection Coefficient	Individual	Age	Clade	Vaccinated
ORF1a	C1218T	S318L	Non	0.4155	103403	42	BA.1	Yes
ORF1b	C14178T	237T	Syn	0.4220	103403	42	BA.1	Yes
ORF1b	G21249T	2594V	Syn	0.3603	102603	46	BA.1	Yes
S	T22579A	D339E	Non	0.3718	105202	54	BA.1	Yes
S	A22905C	L461I	Non	0.4369	103403	42	BA.1	Yes
S	C22943A	N448T	Non	0.4431	105901	49	BA.1	Yes
S	G23282A	D574N	Non	0.3868	103404	36	BA.1	Yes
S	G23282A	D574N	Non	0.4181	102801	39	BA.1	Yes
S	C23987A	P809T	Non	0.3708	107201	50	BA.1	Yes
S	C24904T	1114I	Syn	0.4392	103801	47	BA.1	No
ORF7a	C27434T	T14I	Non	0.4143	103801	47	BA.1	No
ORF8	C28054T	S54L.	Non	0.4009	101703	10	Delta	No
N	C29370T	T366I	Non	0.2828	104501	10	BA.1	Yes
NA	G29742A	NA	NA	0.3544	106103	36	BA.1	Yes

Three of the selected spike amino acid substitutions were in the RBD (Receptor Binding Domain). Outside of the RBD, two individuals shared the positively selected substitution, S:D574N. A third individual had S:D574N in 4 specimens, yet without a positive selection coefficient. None of the iSNV in future lineage defining codons had a positive selection coefficient. However, one individual had both an iSNV in a lineage defining codon (S:547) and an iSNV with a positive selection coefficient in the viral replicase (ORF8:S54L). All of the nonsynonymous spike iSNV were in vaccinated individuals.

We used the SARS-CoV-2 nextstrain build to determine whether any of the iSNV with positive selection coefficients were also identified as increasing in global frequency [37]. None of the iSNV or resulting amino acid changes reached more than 5% globally. The selection coefficients we estimated were only weakly related to the between host selection coefficients estimated by Bloom and Neher [38] (S7 Fig).

## Discussion

In this intensive evaluation of serially sampled individuals in a longitudinal household transmission study, we found that within-host SARS-CoV-2 populations are dominated by purifying selection and genetic drift. This results in low levels of diversity and low rates of divergence, consistent with previous studies [8–10,39,40]. There were differences in divergence rate based on age and in the frequency of iSNV based on vaccination, but these are unlikely to be biologically significant and are not necessarily causative. The lack of biologically meaningful differences between vaccinated and unvaccinated individuals may partially be the result of prior infection in the unvaccinated group. Multiple factors influenced the number of iSNV per specimen, notably day of sampling. Positive selection was rare, but when present, it tended to be enriched in key areas of spike and the RBD.

The low level of diversity is similar to what we and others have reported for SARS-CoV-2 [8–10,39,40]. Some study-specific differences in diversity are noteworthy. For example, Farjo *et al.* (with specimens from 40 individuals) observed higher numbers of iSNV in vaccinated individuals, while we found higher numbers of iSNV in unvaccinated individuals [11]. However, their quality metrics differed between vaccinated and unvaccinated individuals, and their sample size was smaller than the present study. Additionally Gu *et al.* found that the number of iSNV per specimen was higher in VOC compared to non-VOC clades, but did not find any differences between VOC clades [12]. In contrast, we found Delta and BA.2 had more iSNV than BA.1. Our frequency threshold for variant calling was lower, and potentially more sensitive to differences in iSNV number. Variation between cohorts likely contributes to differences between studies, but different study designs and methods also account for dissimilarities.

SARS-CoV-2 has comparable within-host dynamics to influenza A virus. The distribution of allele frequencies is very similar in influenza A and SARS-CoV-2, with most iSNV found at very low frequencies [24,41,42]. However, compared to studies of influenza with the same iSNV threshold, SARS-CoV-2 had fewer iSNV per specimen despite the genome being twice the size. SARS-CoV-2 also had lower divergence rates of 10^-6^ div/site/day for synonymous sites and 10^-7^ for nonsynonymous sites, compared to 10^-5^ and 10^-6^ for influenza A in synonymous and nonsynonymous sites respectively [24,41]. The lower within-host diversity of SARS-CoV-2 is largely attributable to the difference in mutation rates. With its proofreading capabilities, SARS-CoV-2 has a mutation rate of 9 x 10^-7^ mutations per nucleotide per replication cycle [43] compared to 2 x 10^-6^ in influenza A (using analogous assays) [44]. The strength of genetic drift may also contribute to the observed differences. While both influenza A virus (Ne ~ 150–300) [41,42] and SARS-CoV-2 have small effective population sizes, the smaller effective population size in SARS-CoV-2 will result in more genetic drift. More of the variation will be lost from the population or not repeatedly sampled due to changes in population structure. These within-host dynamics are largely consistent with the neutral theory of evolution [45]. Strongly deleterious mutations are removed quickly from the population and the remaining variation is largely neutral.

Despite overall similar patterns of within-host dynamics between SARS-CoV-2 and influenza A virus, there are differences in the nature of selected sites. In influenza A virus, we have not found an overrepresentation of selected sites in hemagglutinin (HA), including antigenic sites, or in neuraminidase (NA) [41]. In contrast, in SARS-CoV-2 we found a greater number of positively selected sites in spike (7/13) and in the RBD (3) than expected by chance. This is consistent with selection for immune escape. Within the RBD, S:D339E was under positive selection. Although this exact amino acid substitution has not previously been known to be under selection, S:339 is the most variable amino acid in spike [46]. Additionally, G339D is a lineage defining mutation in BA.1, BA.2, BA.4, and BA.5 [47], and D339H is a lineage defining mutation for BA.2.75, XBB, and BA.2.86 [48,49]. Both of these amino acid substitutions have been shown to escape neutralizing antibodies [50,51].

In the RBD, S:448 is an epitope targeted by multiple monoclonal antibodies, including bebtelovimab, imdevimab, and cilgavimab [47]. These monoclonal antibodies have high similarity to germline encoded antibodies [52–54], making S:448 an epitope that is likely to be commonly targeted across individuals. Outside of the RBD, two individuals in different households had D574N under positive selection. This substitution has been observed in a long-term infection of an immunocompromised patient [55] and also detected in a small proportion of BA.5 lineages. Mutations at sites 371, 339, and 574 have all been shown to affect the propensity of the RBD to adopt a down versus up conformation, which can reduce neutralization by polyclonal serum antibodies by reducing antibody binding to RBD epitopes that are only accessible in the up-RBD conformation [56–58].

This infrequent but detectable positive selection may be due to the timing of these infections relative to viral emergence. This study enrolled individuals within approximately the first 18–24 months of the pandemic. At this time, only the Wuhan strain spike was used for vaccination, leading to a relatively narrow antibody repertoire. A narrow antibody repertoire may cause uniform selection pressure, with one or a few mutations being sufficient for SARS-CoV-2 to be resistant to a majority of the host antibodies, similar to treatment with monoclonal antibodies [52,59]. In our study, six of the selected sites in spike, all of the nonsynonymous sites, and all of the selected sites in the RBD occurred in vaccinated individuals. Over time as the number of exposures and lineages individuals are exposed to increases, their antibody repertoires also increase [60,61]. As the antibody repertoire diversifies, individual mutations may make SARS-CoV-2 resistant to only a small proportion of antibodies, leading to weaker selection [61]. Earlier in the pandemic there may have been low levels of selection due to lack of even partial immunity, coinciding with a period of global evolutionary stasis [43].

Despite finding immunologically relevant iSNV, our results had low predictive power for trends in SARS-CoV-2 evolution globally. None of the iSNV under positive selection or the corresponding amino acid substitutions reached >5% frequency globally at any time. Two individuals with BA.1 infections had a lineage defining mutation, S:371F, for BA.2. However, the mutation remained at very low frequencies within these two individuals. In the first individual, the selection coefficient was not statistically significantly different than 0 (s = -0.07), and a selection coefficient was unable to be calculated for the second individual due to the number of specimens. With low effective population sizes and stochastic dynamics, our estimates of positive selection are conservative. However, combining within-host variant data with other sources (e.g., deep mutational scanning or inferred between-host selection coefficients) may be fruitful for understanding the evolutionary trajectory of SARS-CoV-2.

A major strength of this study is daily sampling, with up to 9 successfully sequenced specimens per individual, allowing us to examine allele trajectories. Summary statistics meant to detect selection can be misleading due to genetic linkage and hitchhiking [13]. These effects are especially prominent in cases where there are strong bottlenecks and low levels of recombination. With serial sampling, we were able to calculate selection coefficients and detect hitchhiking of synonymous mutations with a physically linked nonsynonymous mutation. To illustrate, in one individual, there were three iSNV with nearly identical allele trajectories: 2 nonsynonymous and 1 synonymous. Most likely, the nonsynonymous iSNV in ORF1a and the synonymous iSNV in ORF1b were swept along with the nonsynonymous iSNV in spike (L461I).

Our study has several limitations. First, our results may not generalize to other phases of the SARS-CoV-2 pandemic. The study took place over 6 months in the second year of the pandemic after the availability of a single vaccine formulation. Results may differ as vaccine and exposure history become more variable across the population and as SARS-CoV-2 has had a longer evolutionary history with human hosts. Indeed, we speculate that SARS-CoV-2 evolution during acute infections could become more similar to the dynamics of influenza A virus within hosts [41,42]. Second, there is always the possibility of inaccurate variant calls. However, this possibility was mitigated by sequencing all specimens in replicate and sequencing multiple specimens per person reduces this possibility. Third, SARS-CoV-2 has significant compartmentalization [62,63], and we are only sampling one location in the body; but when compared, nasal and saliva specimens have similar within-host dynamics dominated by stochastic processes [11]. Fourth, the bias towards symptomatic index cases may reduce the generalizability. Although contact cases had both symptomatic and asymptomatic infections included.

Across studies, acute respiratory viruses have similar within-host dynamics: tight bottlenecks, low genetic diversity, and populations dominated by purifying selection and genetic drift [8–12,19,39–42,64,65]. Overall, our findings are consistent with this pattern. However, nuanced differences exist between viruses, cohorts, and demographic features. In our cohort, within-host positive selection was rare, but appeared to frequently be immune mediated when present. As viruses adapt to human hosts and the population develops immunity, it will be important to follow the shifting impacts on within-host dynamics and selective pressure.

## Supporting information

S1 Table Demographic information and infection details for individuals in this study (n = 105). (PDF)

S2 Table Comparisons of the number of iSNV per specimen and of iSNV frequency. For statistically significant differences the p values are bolded. χ^2^ test statistics are from Kruskal-Wallis rank sum tests and W test statistics are from Mann-Whitney U tests.
(PDF)

S3 Table Effects of viral load. Statistically significant differences are bolded. Z test statistics are from comparisons of iSNV number and iSNV frequency. T test statistics are from comparisons with divergence rate.
(PDF)

S4 Table Comparisons of divergence rates. Statistically significant differences are bolded. χ^2^ test statistics are from Kruskal-Wallis rank sum tests, and W test statistics are from Mann-Whitney U tests.
(PDF)

S5 Table Post hoc (Dunn) tests for divergence rate between genes using a per scpeimen estimate. Statistically significant differences are bolded for the adjusted P values.
(PDF)

S6 Table Post hoc (Dunn) tests for divergence rate of synonymous mutations between genes using linear regressions. Statistically significant differences are bolded for the adjusted P values.
(PDF)

S1 Fig Trajectories of viral load by individual over the course of their infection. (PDF)

S2 Fig Sequencing coverage (A) Boxplots of coverage across the genome in non-overlapping windows of 400 bp for specimens with high quality sequencing. The box shows the first quartile, median, and third quartile. The whiskers are 1.5x interquartile range, and the dots are the outliers. **(B)** The number of iSNV per sample by average sequencing depth. iSNV = intra-host single nucleotide variants.
(PDF)

S3 Fig iSNV frequency. **(A)** mutation type, **(B)** vaccination status, **(C)** age with child <18 and adult 18 + , **(D)** clade, and **(E)** days post symptom onset. The red lines are the mean. iSNV = intra-host single nucleotide variants.
(PDF)

S4 Fig Viral load and iSNV dynamics. Effects of viral load on **(A)** iSNV frequency, **(B)** iSNV number per specimen, and **(C)** divergence rates. Green is synonymous, and purple is nonsynonymous. iSNV = intra-host single nucleotide variants.
(PDF)

S5 Fig Number of specimens collected per day post symptom onset. **(A)** vaccination status, **(B)** age with child <18 and adult 18 + , and **(C)** clade.
(PDF)

S6 Fig Divergence rate (divergence/site/day) using linear regressions. **(A)** vaccination status, **(B)** age with child <18 and adult 18 + , **(C)** clade, and **(D)** gene (green synonymous, purple nonsynonymous).
(PDF)

S7 Fig Comparison of the within-host selection coefficient and the population level selection coefficient for Bloom & Neher 2023. Green is synonymous and purple is nonsynonymous. Triangles are mutations in spike, and circles are in non-spike genes.
(PDF)
