# Supplementary material for: In depth sequencing of a serially sampled household cohort reveals the within-host dynamics of Omicron SARS-CoV-2 and rare selection of novel spike variants
Source: PLoS Pathog. 2025 Apr 28;21(4):e1013134. doi: 10.1371/journal.ppat.1013134 (PMC12074595; doi:10.1371/journal.ppat.1013134)
Supplement: S1 Table — (PDF) [file ppat.1013134.s001.pdf]

Table S1. Demographic information and infection details for individuals in this study (n=105).

Characteristic		n
**Age**	Child (<18 years)	32
	Adult (≥18 years)	73
**Clade**	Delta	17
	BA.1	86
	BA.2	2
**Vaccination**	Yes	76
	No	23
	Missing	6
**Sex**	Female	57
	Male	47
	Missing	1
**Symptomatic**	Yes	103
	No	1
	Missing	1
**Multiple specimens sequenced**	Yes	99
	No	6
