# Supplementary material for: In depth sequencing of a serially sampled household cohort reveals the within-host dynamics of Omicron SARS-CoV-2 and rare selection of novel spike variants
Source: PLoS Pathog. 2025 Apr 28;21(4):e1013134. doi: 10.1371/journal.ppat.1013134 (PMC12074595; doi:10.1371/journal.ppat.1013134)
Supplement: S2 Table — For statistically significant differences the p values are bolded. χ2 test statistics are from Kruskal-Wallis rank sum tests and W test statistics are from Mann-Whitney U tests. (PDF) [file ppat.1013134.s002.pdf]

S2 Table. Comparisons of the number of iSNV per specimen and of iSNV frequency. For statistically significant differences the p values are bolded. $\chi^2$ test statistics are from Kruskal-Wallis rank sum tests and W test statistics are from Mann-Whitney U tests.

| **Number of iSNV per sample** | Test statistic ($\chi^2$ or W) | df | p value |
|---|---|---|---|
| Age | 28066 | 1 | **0.011** |
| Vaccination | 37242 | 1 | **< 0.001** |
| Days Post Symptom Onset | 35.768 | 17 | **0.005** |
| Clade | 38.751 | 2 | **< 0.001** |
| | | | |
| ***Clade - Post Hoc*** | Test statistic (Z) | p.unadj | p.adj |
| Delta vs BA.1 | 5.890058 | **< 0.001** | **< 0.001** |
| Delta vs BA.2 | -0.278536 | 0.781 | 0.781 |
| BA.1 vs BA.2 | -2.392985 | **0.017** | **0.033** |
| | | | |
| **iSNV Frequency** | Test statistic ($\chi^2$ or W) | df | p value |
| Age | 144856 | 1 | 0.792 |
| Vaccination | 135509 | 1 | **0.022** |
| Clade | 2.4914 | 2 | 0.288 |
| Days Post Symptom Onset | 34.069 | 17 | **0.002** |
