# Supplementary material for: In depth sequencing of a serially sampled household cohort reveals the within-host dynamics of Omicron SARS-CoV-2 and rare selection of novel spike variants
Source: PLoS Pathog. 2025 Apr 28;21(4):e1013134. doi: 10.1371/journal.ppat.1013134 (PMC12074595; doi:10.1371/journal.ppat.1013134)
Supplement: S3 Table — Statistically significant differences are bolded. Z test statistics are from comparisons of iSNV number and iSNV frequency. T test statistics are from comparisons with divergence rate. (PDF) [file ppat.1013134.s003.pdf]

S3 Table. Effects of viral load. Statistically significant differences are bolded. Z test statistics are from comparisons of iSNV number and iSNV frequency. T test statistics are from comparisons with divergence rate.

	Estimate	Std. Error	Test statistic (z or t)	p value
Number of iSNV	-0.0023	0.02102	-0.109	0.913
Frequency of ISNV	-0.1719	0.1044	-1.646	0.0997
Divergence rate of nonsynonymous sites	-1.45E-07	1.52E-07	-0.952	0.344
Divergence rate of synonymous sites	-1.02E-07	4.21E-07	-0.242	0.809
