# Supplementary material for: In depth sequencing of a serially sampled household cohort reveals the within-host dynamics of Omicron SARS-CoV-2 and rare selection of novel spike variants
Source: PLoS Pathog. 2025 Apr 28;21(4):e1013134. doi: 10.1371/journal.ppat.1013134 (PMC12074595; doi:10.1371/journal.ppat.1013134)
Supplement: S4 Table — Statistically significant differences are bolded. χ2 test statistics are from Kruskal-Wallis rank sum tests, and W test statistics are from Mann-Whitney U tests. (PDF) [file ppat.1013134.s004.pdf]

S4 Table. Comparisons of divergence rates. Statistically significant differences are bolded. χ^2 test statistics are from Kruskal-Wallis rank sum tests, and W test statistics are from Mann-Whitney U tests.

	**Nonsynonymous**			**Synonymous**		
Point Estimate	Test statistic (χ^2 or W)	df	p value	Test statistic (χ^2 or W)	df	p value
Gene	76.888	8	**<0.001**	55.212	8	**<0.001**
Age	821.5	1	**0.019**	957.5	1	0.165
Days Post Symptom Onset	21.724	17	0.196	15.159	17	0.584
Vaccination	1040.5	1	0.835	1034	1	0.869
Clade	1.6218	2	0.444	3.095	2	0.213
<u>Linear Regression</u>						
Gene	6.2674	8	0.617	27.036	8	**<0.001**
Age	970	1	0.930	996	1	0.769
Vaccination	1152.5	1	0.051	873	1	0.751
Clade	1.375	2	0.503	0.036423	2	0.982
