# Supplementary material for: In depth sequencing of a serially sampled household cohort reveals the within-host dynamics of Omicron SARS-CoV-2 and rare selection of novel spike variants
Source: PLoS Pathog. 2025 Apr 28;21(4):e1013134. doi: 10.1371/journal.ppat.1013134 (PMC12074595; doi:10.1371/journal.ppat.1013134)
Supplement: S5 Table — Statistically significant differences are bolded for the adjusted P values. (PDF) [file ppat.1013134.s005.pdf]

S5 Table. Post hoc (Dunn) tests for divergence rate between genes using a per specimen estimate. Statistically significant differences are bolded for the adjusted P values.

Nonsynonymous					Synonymous		
Comparison		Z	P.unadj	P.adj	Z	P.unadj	P.adj
M	N	-0.804078	0.421	1.000	-1.1911933	0.234	1.000
M	ORF1a	-6.5913858	< 0.001	**< 0.001**	-4.3091874	< 0.001	**< 0.001**
N	ORF1a	-5.7873077	< 0.001	**< 0.001**	-3.1179941	0.002	0.046
M	ORF1b	-3.5232883	< 0.001	**0.012**	-4.6389208	< 0.001	**< 0.001**
N	ORF1b	-2.7192103	0.007	0.144	-3.4477275	0.001	**0.015**
ORF1a	ORF1b	3.06809744	0.002	0.056	-0.3297334	0.742	1.000
M	ORF3a	-0.5484495	0.583	1.000	-0.595282	0.552	1.000
N	ORF3a	0.25562853	0.798	1.000	0.59591129	0.551	1.000
ORF1a	ORF3a	6.04293627	< 0.001	**< 0.001**	3.71390542	< 0.001	**0.006**
ORF1b	ORF3a	2.97483883	0.003	0.073	4.04363881	< 0.001	**0.002**
M	ORF6	-0.0066613	0.995	0.995	-0.3089677	0.757	1.000
N	ORF6	0.79741668	0.425	1.000	0.88222558	0.378	1.000
ORF1a	ORF6	6.58472442	< 0.001	**< 0.001**	4.00021971	< 0.001	**0.002**
ORF1b	ORF6	3.51662698	< 0.001	**0.012**	4.3299531	< 0.001	**< 0.001**
ORF3a	ORF6	0.54178815	0.588	1.000	0.28631429	0.775	1.000
M	ORF7a	-1.6364667	0.102	1.000	0.00377557	0.997	1.000
N	ORF7a	-0.8323887	0.405	1.000	1.19496889	0.232	1.000
ORF1a	ORF7a	4.95491909	< 0.001	**< 0.001**	4.31296302	< 0.001	**< 0.001**
ORF1b	ORF7a	1.88682164	0.059	1.000	4.64269641	< 0.001	**< 0.001**
ORF3a	ORF7a	-1.0880172	0.277	1.000	0.5990576	0.549	1.000
ORF6	ORF7a	-1.6298053	0.103	1.000	0.31274331	0.754	1.000
M	ORF8	-0.2756125	0.783	1.000	0.00629262	0.995	1.000
N	ORF8	0.52846549	0.597	1.000	1.19748594	0.231	1.000
ORF1a	ORF8	6.31577323	< 0.001	**< 0.001**	4.31548007	< 0.001	**0.001**
ORF1b	ORF8	3.24767579	0.001	**0.031**	4.64521345	< 0.001	**< 0.001**
ORF3a	ORF8	0.27283696	0.785	1.000	0.60157465	0.547	1.000
ORF6	ORF8	-0.2689512	0.788	1.000	0.31526036	0.753	1.000
ORF7a	ORF8	1.36085415	0.174	1.000	0.00251705	0.998	0.998
M	S	-2.8735311	0.004	0.097	-2.3660258	0.018	0.396
N	S	-2.0694531	0.039	0.732	-1.1748325	0.240	1.000
ORF1a	S	3.71785465	< 0.001	**< 0.001**	1.94316163	0.052	0.988
ORF1b	S	0.6497572	0.516	1.000	2.27289502	0.023	0.484
ORF3a	S	-2.3250816	0.020	0.401	-1.7707438	0.077	1.000
ORF6	S	-2.8668698	0.004	0.095	-2.0570581	0.040	0.794
ORF7a	S	-1.2370644	0.216	1.000	-2.3698014	0.018	0.409
ORF8	S	-2.5979186	0.009	0.197	-2.3723184	0.018	0.424
