# Supplementary material for: In depth sequencing of a serially sampled household cohort reveals the within-host dynamics of Omicron SARS-CoV-2 and rare selection of novel spike variants
Source: PLoS Pathog. 2025 Apr 28;21(4):e1013134. doi: 10.1371/journal.ppat.1013134 (PMC12074595; doi:10.1371/journal.ppat.1013134)
Supplement: S6 Table — Statistically significant differences are bolded for the adjusted P values. (PDF) [file ppat.1013134.s006.pdf]

S6 Table. Post hoc (Dunn) tests for divergence rate of synonymous mutations between genes using linear regressions. Statistically significant differences are bolded for the adjusted P values.

Comparison		Z	P.unadj	P.adj
M	N	1.504	0.132	1
M	ORF1a	0.846	0.397	1
N	ORF1a	2.350	0.018	0.525
M	ORF1b	1.972	0.049	1
N	ORF1b	3.476	<0.001	**0.018**
ORF1a	ORF1b	1.126	0.260	1
M	ORF3a	0.931	0.352	1
N	ORF3a	0.573	0.567	1
ORF1a	ORF3a	1.777	0.075	1
ORF1b	ORF3a	2.903	0.004	0.118
M	ORF6	0.280	0.780	1
N	ORF6	1.225	0.221	1
ORF1a	ORF6	1.126	0.260	1
ORF1b	ORF6	2.251	0.024	0.633
ORF3a	ORF6	0.652	0.515	1
M	ORF7a	0.963	0.335	1
N	ORF7a	0.541	0.589	1
ORF1a	ORF7a	1.809	0.070	1
ORF1b	ORF7a	2.935	0.003	0.110
ORF3a	ORF7a	0.032	0.974	0.974
ORF6	ORF7a	0.684	0.494	1
M	ORF8	1.731	0.083	1
N	ORF8	0.227	0.820	1
ORF1a	ORF8	2.578	0.010	0.298
ORF1b	ORF8	3.703	<0.001	**0.008**
ORF3a	ORF8	0.800	0.424	1
ORF6	ORF8	1.452	0.147	1
ORF7a	ORF8	0.768	0.442	1
M	S	1.392	0.164	1
N	S	2.897	0.004	0.117
ORF1a	S	0.546	0.584	1
ORF1b	S	0.579	0.562	1
ORF3a	S	2.324	0.020	0.543
ORF6	S	1.672	0.094	1
ORF7a	S	2.356	0.018	0.536
ORF8	S	3.124	0.002	0.061
