# Supplementary figures and images for: In depth sequencing of a serially sampled household cohort reveals the within-host dynamics of Omicron SARS-CoV-2 and rare selection of novel spike variants
Source: PLoS Pathog. 2025 Apr 28;21(4):e1013134. doi: 10.1371/journal.ppat.1013134 (PMC12074595; doi:10.1371/journal.ppat.1013134)

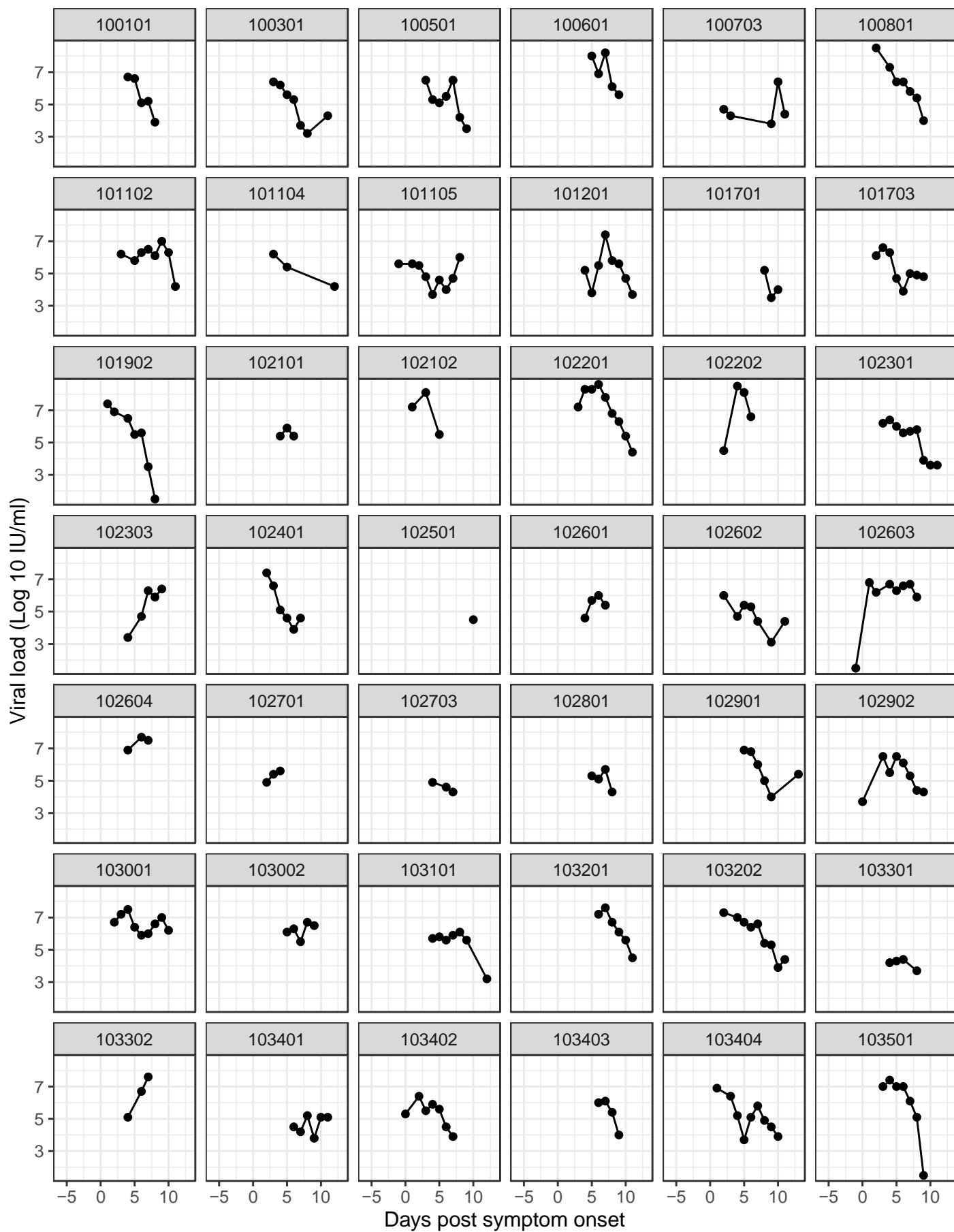

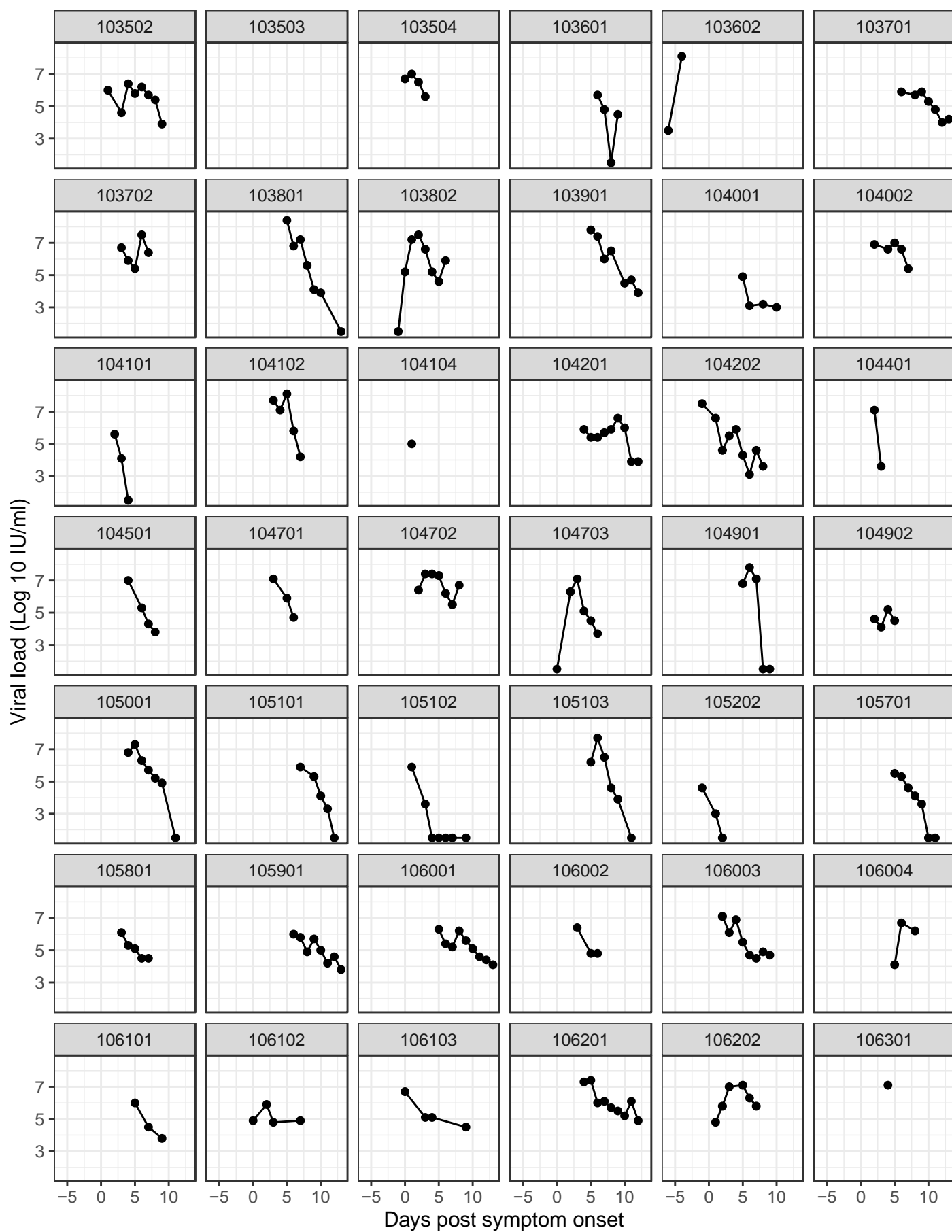

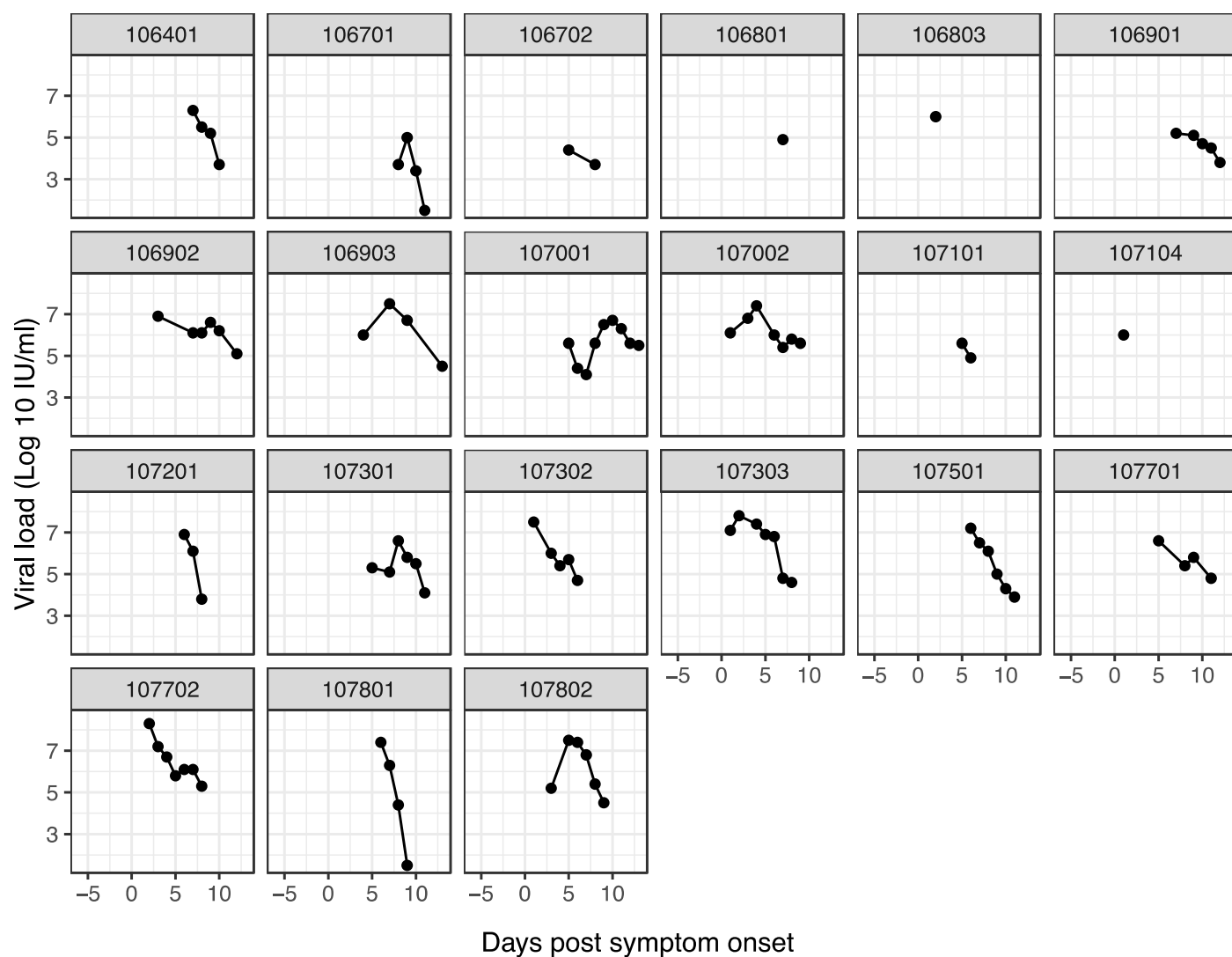

S1 Fig. Trajectories of viral load by individual over the course of their infection

Supplement: S1 Fig — (PDF) [file ppat.1013134.s007.pdf]
