# Supplementary material for: In depth sequencing of a serially sampled household cohort reveals the within-host dynamics of Omicron SARS-CoV-2 and rare selection of novel spike variants
Source: PLoS Pathog. 2025 Apr 28;21(4):e1013134. doi: 10.1371/journal.ppat.1013134 (PMC12074595; doi:10.1371/journal.ppat.1013134)
Supplement: S2 Fig — The box shows the first quartile, median, and third quartile. The whiskers are 1.5x interquartile range, and the dots are the outliers. (B) The number of iSNV per sample by average sequencing depth. iSNV = intra-host single nucleotide variants. (PDF) [file ppat.1013134.s008.pdf]

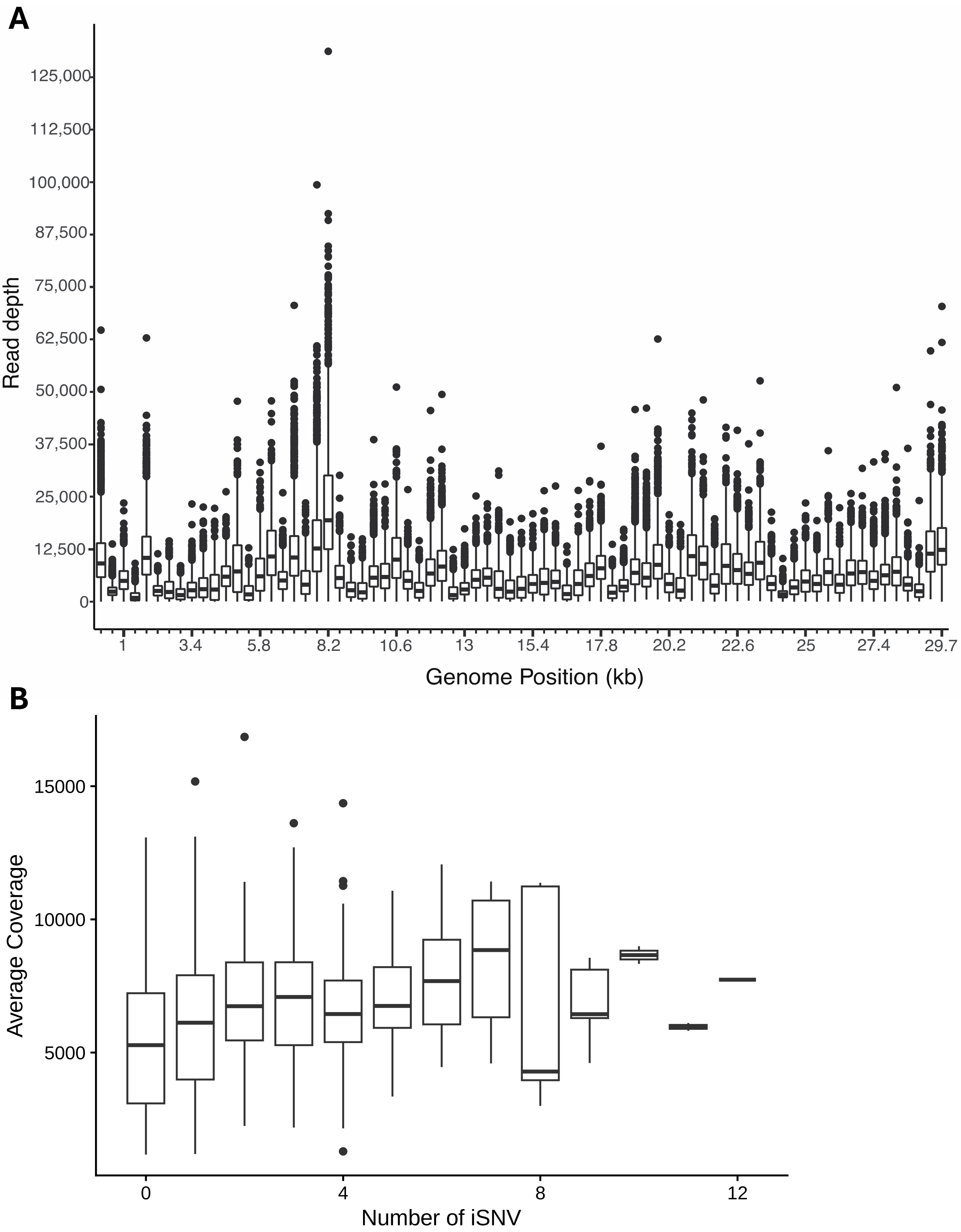

S2 Fig. Sequencing coverage **(A)** Boxplots of coverage across the genome in non-overlapping windows of 400 bp for specimens with high quality sequencing. The box shows the first quartile, median, and third quartile. The whiskers are 1.5x interquartile range, and the dots are the outliers. **(B)** The number of iSNV per sample by average sequencing depth. iSNV = intra-host single nucleotide variants.
