# Supplementary material for: In depth sequencing of a serially sampled household cohort reveals the within-host dynamics of Omicron SARS-CoV-2 and rare selection of novel spike variants
Source: PLoS Pathog. 2025 Apr 28;21(4):e1013134. doi: 10.1371/journal.ppat.1013134 (PMC12074595; doi:10.1371/journal.ppat.1013134)
Supplement: S3 Fig — (A) mutation type, (B) vaccination status, (C) age with child <18 and adult 18 + , (D) clade, and (E) days post symptom onset. The red lines are the mean. iSNV = intra-host single nucleotide variants. (PDF) [file ppat.1013134.s009.pdf]

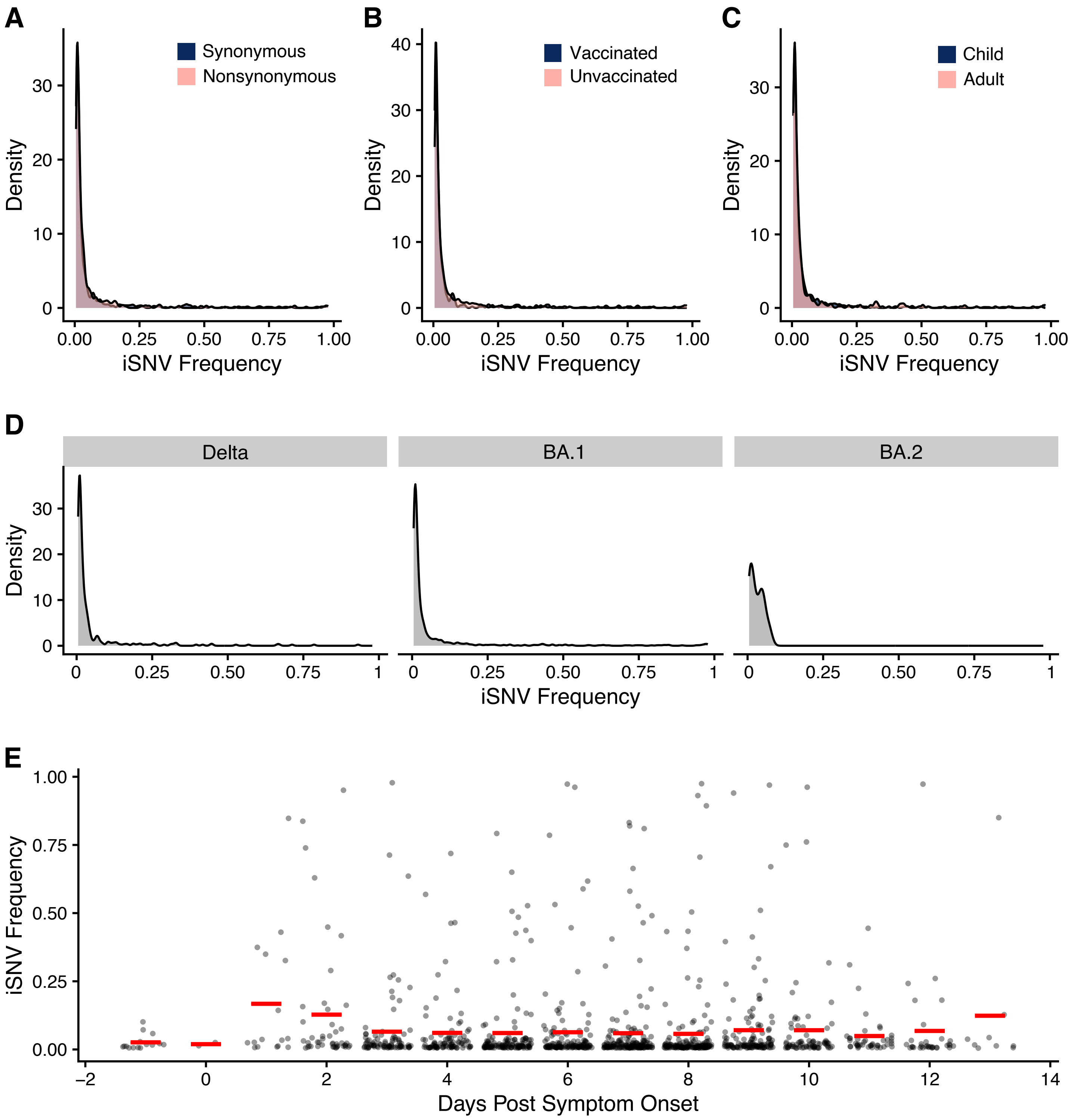

S3 Fig. iSNV frequency. **(A)** mutation type, **(B)** vaccination status, **(C)** age with child <18 and adult 18+, **(D)** clade, and **(E)** days post symptom onset. The red lines are the mean. iSNV = intra-host single nucleotide variants.
