# Supplementary material for: In depth sequencing of a serially sampled household cohort reveals the within-host dynamics of Omicron SARS-CoV-2 and rare selection of novel spike variants
Source: PLoS Pathog. 2025 Apr 28;21(4):e1013134. doi: 10.1371/journal.ppat.1013134 (PMC12074595; doi:10.1371/journal.ppat.1013134)
Supplement: S4 Fig — Effects of viral load on (A) iSNV frequency, (B) iSNV number per specimen, and (C) divergence rates. Green is synonymous, and purple is nonsynonymous. iSNV = intra-host single nucleotide variants. (PDF) [file ppat.1013134.s010.pdf]

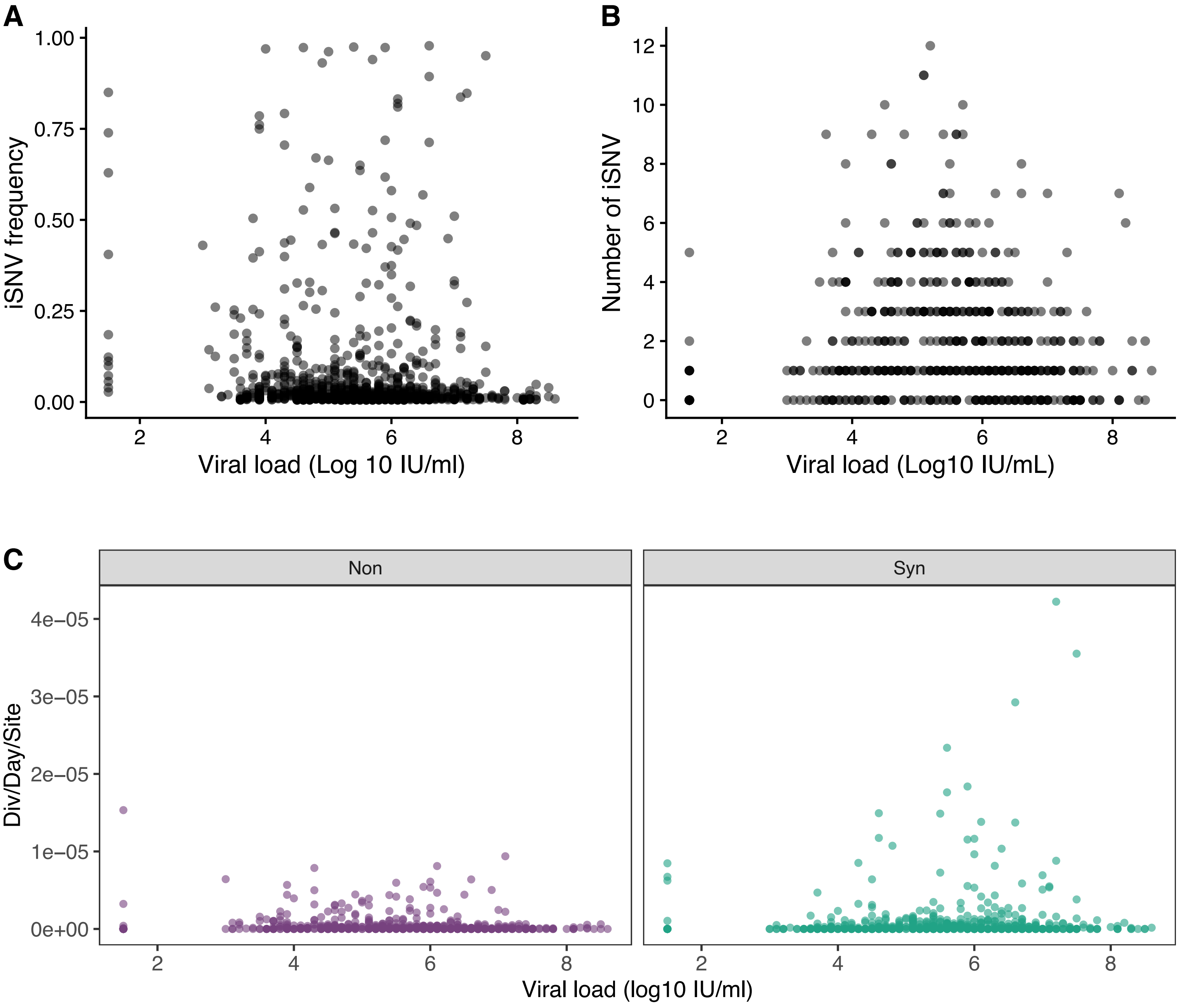


S4 Fig. Viral load and iSNV dynamics. Effects of viral load on **(A)** iSNV frequency, **(B)** iSNV number per specimen, and **(C)** divergence rates. Green is synonymous, and purple is nonsynonymous. iSNV = intra-host single nucleotide variants.
