# Supplementary material for: In depth sequencing of a serially sampled household cohort reveals the within-host dynamics of Omicron SARS-CoV-2 and rare selection of novel spike variants
Source: PLoS Pathog. 2025 Apr 28;21(4):e1013134. doi: 10.1371/journal.ppat.1013134 (PMC12074595; doi:10.1371/journal.ppat.1013134)
Supplement: S5 Fig — (A) vaccination status, (B) age with child <18 and adult 18 + , and (C) clade. (PDF) [file ppat.1013134.s011.pdf]

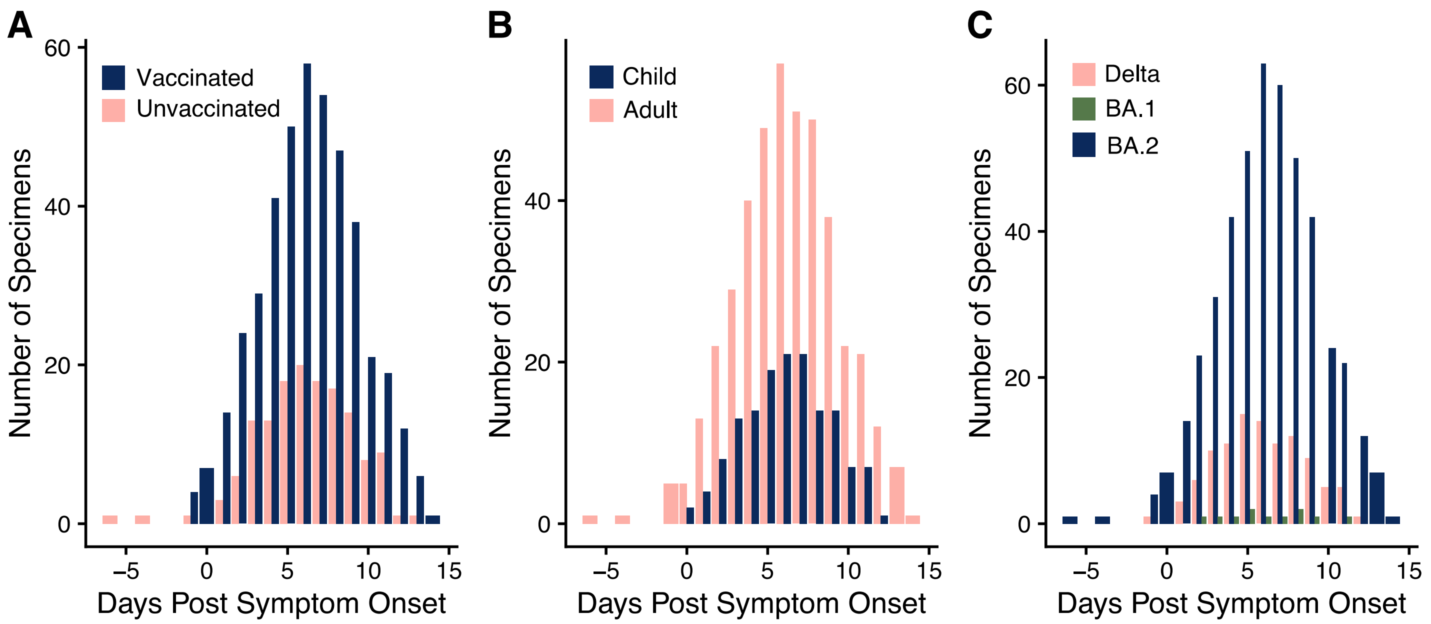

S5 Fig. Number of specimens collected per day post symptom onset. **(A)** vaccination status, **(B)** age with child <18 and adult 18+, and **(C)** clade.
