# Supplementary material for: In depth sequencing of a serially sampled household cohort reveals the within-host dynamics of Omicron SARS-CoV-2 and rare selection of novel spike variants
Source: PLoS Pathog. 2025 Apr 28;21(4):e1013134. doi: 10.1371/journal.ppat.1013134 (PMC12074595; doi:10.1371/journal.ppat.1013134)
Supplement: S6 Fig — (A) vaccination status, (B) age with child <18 and adult 18 + , (C) clade, and (D) gene (green synonymous, purple nonsynonymous). (PDF) [file ppat.1013134.s012.pdf]

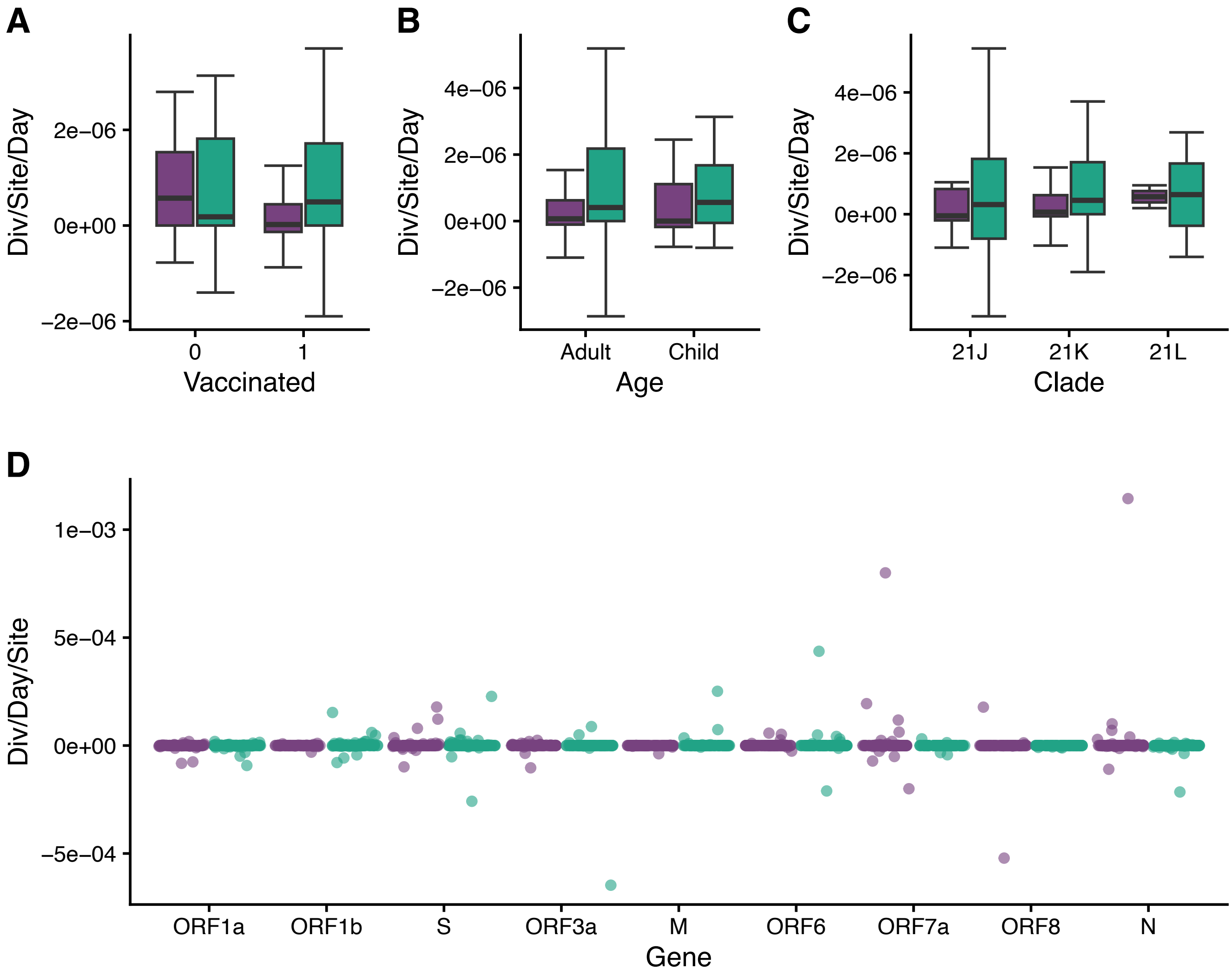

S6 Fig. Divergence rate (divergence/site/day) using linear regressions. **(A)** vaccination status, **(B)** age with child <18 and adult 18+, **(C)** clade, and **(D)** gene (green synonymous, purple nonsynonymous).
