# Supplementary material for: In depth sequencing of a serially sampled household cohort reveals the within-host dynamics of Omicron SARS-CoV-2 and rare selection of novel spike variants
Source: PLoS Pathog. 2025 Apr 28;21(4):e1013134. doi: 10.1371/journal.ppat.1013134 (PMC12074595; doi:10.1371/journal.ppat.1013134)
Supplement: S7 Fig — Green is synonymous and purple is nonsynonymous. Triangles are mutations in spike, and circles are in non-spike genes. (PDF) [file ppat.1013134.s013.pdf]

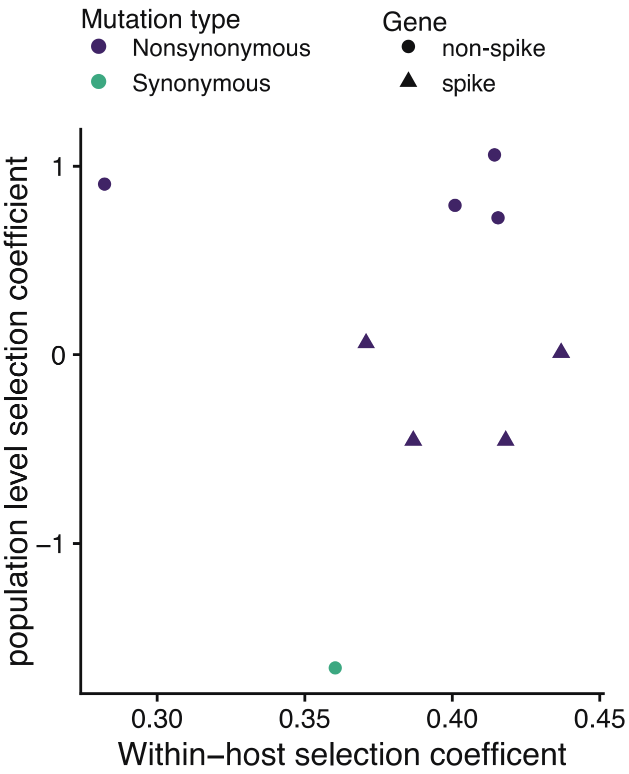


S7 Fig. Comparison of the within-host selection coefficient and the population level selection coefficient for Bloom & Neher 2023. Green is synonymous and purple is nonsynonymous. Triangles are mutations in spike z circles are in non-spike genes.
